# PI3K and MAPK pathways mediate the BDNF/TrkB-increased metastasis in neuroblastoma

**DOI:** 10.1007/s13277-016-5433-z

**Published:** 2016-10-17

**Authors:** Zhongyan Hua, Xiao Gu, Yudi Dong, Fei Tan, Zhihui Liu, Carol J. Thiele, Zhijie Li

**Affiliations:** 1 Medical Research Center, Shengjing Hospital of China Medical University, #36 Sanhao Street, Heping District, Shenyang, 110004 China; 2 Department of Oncology, Shengjing Hospital of China Medical University, Shenyang, China; 3 Department of Neurology, Shengjing Hospital of China Medical University, Shenyang, China; 4 Cellular and Molecular Biology Section, Pediatric Oncology Branch, National Cancer Institute, National Institutes of Health, Bethesda, MD USA

**Keywords:** Neuroblastoma, BDNF, TrkB, Metastasis

## Abstract

Brain-derived neurotrophic factor (BDNF) and its tyrosine kinase receptor TrkB have been reported to be associated with poor prognosis in neuroblastoma (NB) patients. Our previous studies indicated that BDNF activation of TrkB induces chemo-resistance through activation of phosphoinositide-3-kinase (PI3K)/Akt pathway. In this study, we investigated the role of BDNF/TrkB on metastasis in NB. A tetracycline-regulated TrkB-expressing NB cell line (TB3) was used. Scratch wound healing assay, Boyden chamber migration, and invasion assays were performed to study the migration and invasion of TB3 cells. A tumor xenograft model using SCID-Beige mice was utilized to detect the metastasis of NB tumors in vivo. Inhibitors of PI3K, MAPK, Akt, and mTOR were used. Western blotting was performed to study the expressions of P-Akt, P-Erk, and P-mTOR. Our results showed that in TrkB-expressing NB cells, BDNF treatment significantly increased gap closing (*P* < 0.01) in scratch wound healing assay, also significantly enhanced the numbers of migrating cells (*P* < 0.01) and invading cells (*P* < 0.01) in the Boyden chamber migration and invasion assays. In vivo, NB distant metastases were significantly increased in mice with TrkB-expressing xenograft tumors compared to those with non-TrkB-expressing tumors (*P* < 0.05). Pre-treatment with any of the inhibitors for PI3K (LY294002), MAPK (PD98059), Akt (perifosine), or mTOR (rapamycin) blocked the BDNF/TrkB-induced increases of cell migration and invasion in TB3 cells, and also blocked the BDNF/TrkB-induced expressions of P-Akt, P-Erk, and P-mTOR. These data indicated that BDNF/TrkB increased metastasis in NB via PI3K/Akt/mTOR and MAPK pathways, and BDNF/TrkB and the downstream targets may be potential targets for the treatment of NB metastasis.

**Electronic supplementary material:**

The online version of this article (doi:10.1007/s13277-016-5433-z) contains supplementary material, which is available to authorized users.

## Introduction

Neuroblastoma (NB) is one of the most common pediatric malignancies that derives from neural crest precursor cells. It is an extracranial solid tumor that most frequently occurs in adrenal gland [1–2]. Tumor spontaneous regression or differentiation happens in patients with low-risk disease, and surgery with little or no adjunctive therapy is effective for these patients [2–3]. While for patients with high-risk diseases, chemo-resistance and metastasis are the two main problems. Patients may initially response to chemotherapy, but chemo-resistance would develop soon. Wide metastasis usually occurs at diagnosis. Despite of multimodality chemotherapy and stem cell transplantation, satisfactory response still could not be achieved. The long-term survival rate of these patients is less than 40 % [2–5]. So, more effective treatments are urgently needed.

Brain-derived neurotrophic factor (BDNF) is a member of neurotrophin family, and it is known as one of key factors for the sympathetic nervous system development [2–3]. Previous reports show that patients with an unfavorable prognosis are more likely to express BDNF and its tyrosine kinase receptor TrkB [6–8]. The association between BDNF/TrkB expression and chemo-resistance in NB has been studied. It was found that BDNF activation of TrkB induced chemo-resistance in NB, and that was mainly mediated by PI3K/Akt signaling pathway. Treatment targeting the PI3K/Akt pathway attenuated the BDNF/TrkB-induced chemo-resistance [9–14]. Although TrkB expression with invasive capability in NB has been reported [15–16], a systemic study of the role of BDNF/TrkB in the NB metastasis is still needed. In this study, we provide evidence supporting the hypothesis that BDNF/TrkB promotes the NB cell metastasis both in vitro and in vivo. We find that the metastasis potential stimulated by activation of the BDNF/TrkB pathway in NB cells is blocked by inhibiting activation of PI3K and MAPK pathways. Furthermore, we find that downstream targets of BDNF/TrkB-stimulated migration and invasion of NB cells may be Akt and mTOR. All of these results suggest that BDNF/TrkB and its downstream targets may be the new targets in the treatment of NB metastasis.

## Materials and methods

### TrkB-expressing cell line and cell culture

A tetracycline (TET)-regulated TrkB-expressing NB cell line TB3 was utilized. In the presence of TET, TrkB expression is suppressed, and in the absence of TET, TrkB expression is induced [9]. TB3 cells were cultured in RPMI 1640 (Bioind, Israel) containing 10 % fetal bovine serum (FBS; Bioind, Israel), 2 mM glutamine, antibiotics, and puromycin (0.5 μg/ml), and in the presence or absence of tetracycline (1 μg/ml) at 37 °С in 5 % CO_2_ incubator.

### Reagents

Recombinant human BDNF was obtained from PeproTech, Inc. (Rocky Hill, NJ, USA). Puromycin and tetracycline were purchased from Sigma Chemical Company, Inc. (St Louis, MO, USA). PI3K inhibitor LY294002, MAPK inhibitor PD98059, and mTOR inhibitor rapamycin were purchased from Cell Signaling Technology (Beverly, MA, USA). Akt inhibitor perifosine was obtained from Selleckchem (Houston, Texas, USA).

### Treatment

To repress TrkB expression, TB3 cells were cultured in the presence of tetracycline (1 μg/ml) for 3 days. To study the blockage of BDNF/TrkB effect, pharmacological inhibitors, LY294002 (10 μM), PD98059 (10 μM), perifosine (5 μM), and rapamycin (100 nM), were pre-treated 1 h before administration of BDNF. TB3 cells were treated with BDNF (100 ng/ml) for 6 h for the migration and 24 h for the invasion assays, and treated for 30 h for the scratch wound healing assays.

### Northern analysis

Total RNA was isolated from TB3 cells or TB3 tumor tissues with RNeasy kit (Qiagen, Dusseldorf, Germany) following the manufacturer’s protocol. Thirty micrograms of total RNA was electrophoresed in 1 % agarose-6 % formaldehyde gels containing 2 mg/ml ethidium bromide, and then blotted to nylon membrane. Hybridization was performed with ^32^P-labeled insert DNA isolated from a plasmid containing TrkB.

### Scratch wound healing assay

TB3 cells were seeded into 24-well tissue culture plate (Corning, NY, USA). After 48 h of growth, the confluent monolayer of the cells was scratched with a 200-μl pipette tip across the center of the well. The cells were washed with media and treated with BDNF with/without inhibitors. The pharmacological inhibitors, LY294002, PD98059, perifosine, and rapamycin, were pre-treated 1 h before administration of BDNF as described above. Cell migration was photographed at 10× magnification at 0 and 30 h after treated with BDNF. The wound widths at the beginning and at the end of the experiments were measured by Image-Pro Plus software respectively. The cell migration rate was calculated as (*B* − *E*) / *B* × 100 %, in which *B* indicates wound width at the beginning of the experiment, and *E* indicates wound width at the end of the experiment. The experiments were repeated three times.

### Boyden chamber migration and invasion assays

The TB3 cells were cultured in 10 % FBS RPMI 1640 media until 60–70 % confluence. The cells were harvested in 5 % FBS RPMI 1640 media and seeded at 100-μl media of 4 × 10^4^/insert for migration assay or 8 × 10^4^/insert for invasion assay. The 24-well Boyden chamber trans-well insert (Corning, NY, USA) has 8.0-μm-pore polyethylene teraphthalate membrane at the bottom, which was pre-coated with 30 μl matrigel (BD, Biosience) (1:3 diluted with plain media) for invasion assay or left uncoated for migration assay. Bottom wells were fed with 600 μl of 15 % FBS RPMI 1640 media. BDNF was placed in the bottom of the well. After 6 or 24 h, the non-migrating or non-invading cells were removed with cotton-tipped applicator by scraping the upperside of the insert. The cells that migrated or invaded to the underside of the insert were stained using hematoxylin and eosin for 5 and 1 min, respectively, at room temperature. Photographs of five random fields were taken, and the number of cells was counted to calculate the average number of cells per well that had migrated or invaded. The experiments were repeated three times. In the experiments using pharmacological inhibitors, LY294002, PD98059, perifosine, and rapamycin, were placed in the upper of the well, and then the assays were conducted as described above.

### Western blotting

TB3 cells were treated with inhibitors and BDNF as described above, then washed twice with cold PBS, and harvested, and total protein was extracted with Whole Cell Lysis Assay (KeyGEN BioTECH) following the manufacturer’s protocol; 30 μg protein in each condition was loaded onto SDS-PAGE gels, transferred to PVDF membrane, and probed with the anti-phospho-Akt (P-Akt, Ser473), anti-phospho-Erk (P-Erk, Thr202/Tyr204), anti-phospho-mTOR (P-mTOR, Ser2481) antibodies (1:1000 dilution, Cell Signaling Tech.), or anti-GAPDH antibody (1:10,000 dilution, Kangchen bio-tech).

### In vivo metastasis study

TB3 cells were cultured in 10 % FBS RPMI-1640, harvested, washed with Hank balanced salt solution (HBSS), and re-suspended in HBSS. A total of 50 μl of cell suspension containing 4 × 10^6^ TB3 cells were implanted into the left gastrocnemius muscle of SCID-Beige mice aged 4–6 weeks (Taconic, Germantown, NY, USA). Mice were given water supplemented with placebo (sucrose) or tetracycline (with sucrose) 1 week before tumor cells injection, and this was continued throughout the experiment. The experiment was stopped when the mice reached the ending criteria (based on their tumor size and whole body status), and mice were euthanized by asphyxiation with regulated CO_2_. Gross metastasis in the brain, chest cavity, and abdominal cavity was evaluated. The adrenal gland, liver, lungs, and brain were harvested, fixed in 10 % formalin, and used for the HE staining.

The animal study was approved by the Animal Care and Use Committee of the National Cancer Institute, and all mouse treatments, including their housing, were in accordance with the institutional guidelines (PB-023).

### Statistical analysis

Comparisons between two groups were performed using the Student’s *t* test. The results were shown as means ± SD. The tumor metastasis in the two groups of mice was compared by Fisher analysis.

## Results

### TET-regulated TrkB-expressing cell line

**Fig. 1:**
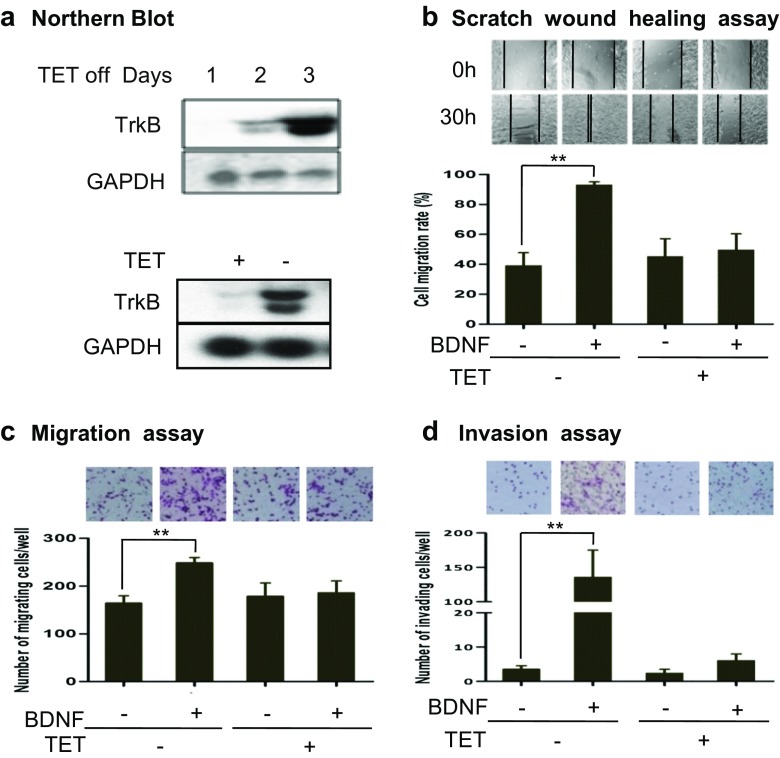
The roles of BDNF/TrkB in TB3 cell migration and invasion in vitro. **a** TET regulated TrkB-expressing cell line. Normally, TB3 cells were cultured with TET (1 μg/ml). To induce the TrkB expression, TET was removed from culture media for 1, 2, and 3 days, and then TB3 cells were harvested and Northern blot was performed. A comparison of TrkB expressions in TB3 cells with or without TET (1 μg/ml, 3 days) was also done. **b**, **c** The role of BDNF/TrkB on TB3 cell migration. Scratch wound healing assay was performed. Cell migration was photographed at 10× magnification at 0 h and 30 h after treated with BDNF. The wound healing width was measured by Image-Pro Plus software. The cell migration rate was calculated as described in “Materials and methods” section. Bars, SD. **P < 0.01, BDNF-treated vs. control (**b**). Migration assay was done as described in “Materials and methods” section. Representative fields of migrating cells under microscope were shown upper of the figure. The cells that migrated to the underside of the inserts were counted, and Student’s *t* test was done. *Bars*, SD. **P < 0.01, BDNF-treated vs. control (**c**). **d** The role of BDNF/TrkB on cell invasion. Invasion assay was performed as described in “Materials and methods” section. Representative fields of invading cells under microscope were shown upper of the figure. The cells that invaded to the underside of the inserts were counted, and Student’s *t* test was done. *Bars*, SD. **P < 0.01 BDNF-treated vs. control. The experiments were repeated three times

In our study, we used a TET-regulated TrkB-expressing cell line, TB3. The presence of TET inhibited the TrkB expression, whereas the absence of TET induced the TrkB expression. Northern blot was used to study the expression of TrkB in TB3 cells in the absence or presence of TET. Normally, TB3 cells were cultured in media with TET (1 μg/ml) to inhibit TrkB expression. To observe the induction of TrkB expression in TB3 cells, TET was removed from culture media for 1, 2, and 3 days, and then TB3 cells were harvested. Results showed that TrkB mRNA level was significantly increased 3 days after TET removal from the culture media (Fig. 1a, upper panel). A comparison of TrkB expressions in TB3 cells with or without TET (1 μg/ml, 3 days) was also performed. The TrkB mRNA level was obviously higher in TB3 cells in the absence of TET compared to that in the presence of TET (Fig. 1a, lower panel). So we cultured TB3 cells without TET for at least 3 days to induce the TrkB expression in our following studies. In our previous study, we have shown that adding BDNF to the cells could activate the tyrosine kinase, and further PI3K and MAPK pathways only in the TrkB-expressing cells (TET^−^), not the non-TrkB-expressing cells (TET^+^) [11]. By using this system, we studied the role of BDNF/TrkB in the metastasis of NB in vitro and in vivo.

### The effect of BDNF/TrkB on the migration and invasion of NB cells

To study the role of BDNF/TrkB on cells migration, we first used the scratch wound healing assay. TB3 cells were cultured in 24-well plates in the presence or absence of TET, and in each well, a line was scratched with pipette tip until the cells reached more than 90 % confluences and then cells were treated with BDNF (100 ng/ml) for 30 h. The cell migration rates, analyzed as described in “Materials and methods” section, were 39.2 % in control cells and 93.0 % in BDNF-treated cells. BDNF treatment significantly increased the gap closing in TrkB-expressing cells (*P* < 0.01) (Fig. 1b). However, in the presence of TET (non-TrkB expression), administration of BDNF to TB3 cells did not significantly change the cell gap by 30 h (*P* = 0.67) (Fig. 1b). There was no statistical difference in closure of cell gaps between TET^+^ and TET^−^ cells in the absence of the BDNF treatment (*P* = 0.51) (Fig. 1b). We also used the Boyden chamber migration assay to study the BDNF/TrkB effect on cell migration. TB3 cells were seeded into the uncoated insert, and cells were treated with BDNF for 6 h in the presence or absence of TET. The cells that migrated to the underside of the inserts were counted and photographed (Fig. 1c). The number of migrating cells increased to 1.5-fold after the BDNF treatment in the TrkB-expressing cells (TET^−^) (*P* < 0.01), while there was no significant change in the number of migrating cells after the BDNF treatment when TrkB expression was inhibited (TET^+^) (Fig. 1c).

The role of BDNF/TrkB in TB3 cell invasion was tested using a Boyden chamber invasion assay in which the inserts were coated with matrigel. In the TrkB-expressing cells (TET^−^), the invading cell number was 54-fold higher in the BDNF-treated cells compared to that in the control cells (*P* = 0.015) (Fig. 1d). No significant increase of invading cells was detected after the BDNF treatment in the TrkB-inhibited cells (TET^+^) (Fig. 1d). These results suggested that BDNF/TrkB increased TB3 cell migration and invasion in vitro.

### TrkB expression in NB xenografts increased the metastasis in vivo

**Fig. 2:**
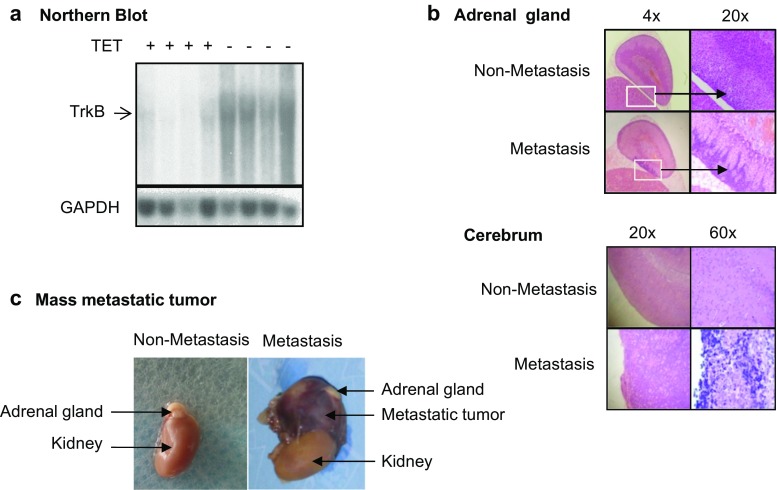
The roles of BDNF/TrkB in NB tumor metastasis in vivo. **a** TET regulated TrkB expression in vivo. Xenograft NB tumor tissues were harvested from SCID-Beige mice, which were fed with or without TET. Animal model was described in “Materials and methods” section. RNA was extracted from the tumor tissues, and TrkB expression was detected by Northern blot. **b**, **c** Metastasis of NB tumors in vivo. The adrenal gland and brain of mice with or without metastasis were demonstrated after HE staining (4× and 20× magnifications for the adrenal glands, and 20× and 60× magnifications for brain tissues) (**b**). A representative metastatic tumor around the adrenal gland was shown (**c**)

Next, we investigated the role of BDNF/TrkB in NB metastasis in vivo. SCID-Beige mice were fed chow with or without TET 1 week before the implantation of TB3 cells until the end of the experiment to inhibit or induce TrkB expression. TB3 cells were implanted into the left gastrocnemius muscle of SCID-Beige mice. To examine the inhibition or induction of TrkB in vivo, we extracted RNA from tumor tissues in the leg and detected TrkB levels by Northern blot, we found that the expression of TrkB mRNA was much higher in TET^−^ tumors compared to that in TET^+^ tumors (Fig. 2a). The metastasis was checked after the mice were euthanized, and tumor metastatic sites were found to be in the adrenal gland, in brain tissue, and in chest cavity. The metastasis rate in mice with TrkB^+^ tumors was 55.6 % (10/18 mice), which was much higher than that in mice with TrkB^−^ tumors (18.2 %, 4/22 mice). A Fisher analysis showed a significant difference in metastasis between the TrkB^+^ and TrkB^−^ groups (*P* = 0.02) (Table 1). Figure 2b showed the microscopic metastasis to the adrenal gland and the brain tissues, and Fig. 2c was the representative mass metastasis around the adrenal gland. These data indicated that expression of TrkB in xenograft NB tumors was more frequently associated with disseminated metastatic spread of NB.

**Table 1:** Comparison of metastasis between TrkB-expressing tumors and non-TrkB-expressing tumors

Group	Metastasis(+)	Metastasis(−)	Total	Ratio of metastasis (%)
TrkB(+)	10	8	18	55.6
TrkB(−)	4	18	22	18.2
Total	14	26	40	35

Fisher’s test: *P* = 0.02

### PI3K and MAPK pathways mediated the BDNF/TrkB-induced increases of cell migration and invasion

We and others previously reported that binding of TrkB and BDNF activates PI3K and MAPK pathways [9–14]. Having found that BDNF/TrkB increased the migration and invasion in vitro and metastasis in vivo, we next investigated the pathway(s) that mediated the BDNF/TrkB-increased metastasis by using pharmacological inhibitors. TB3 cells cultured in the absence of TET were pre-treated with PI3K inhibitor LY294002, or MAPK inhibitor PD98059 for 1 h, and then treated with BDNF for 30 h in scratch wound healing assay, for 6 h in the Boyden chamber migration assay, and for 24 h in Boyden chamber invasion assay.

**Fig. 3:**
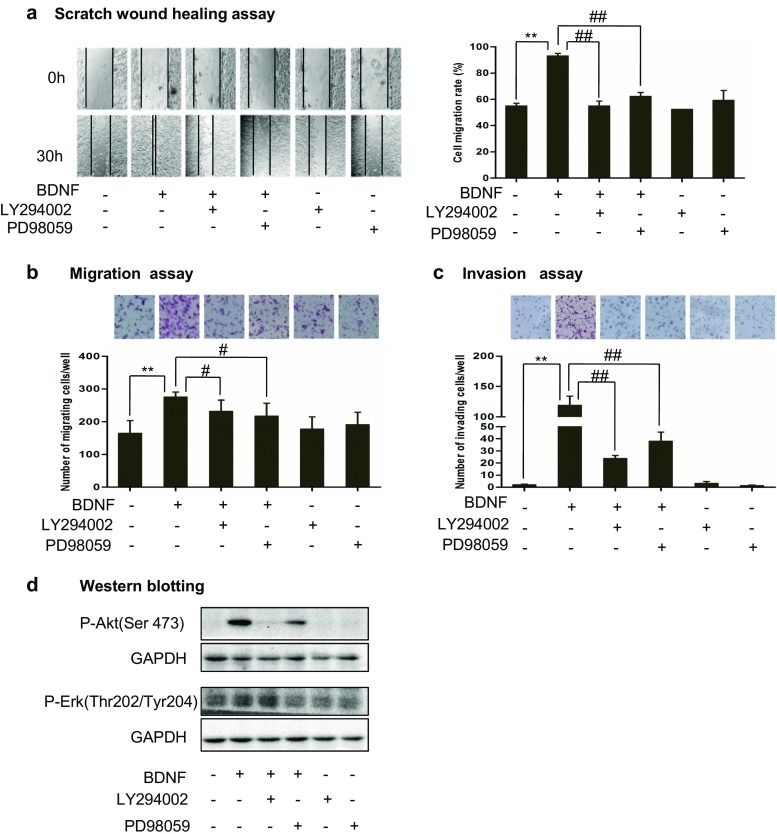
The signal transduction pathways that mediate BDNF/TrkB effects on cell migration and invasion. **a** TB3 cells cultured in the absence of TET were scratched with a 200-μl pipette tip across the center of the well, and then cells were pre-treated with PI3K inhibitor LY294002 or MAPK inhibitor PD98059 for 1 h followed by the BDNF treatment for 30 h. Gap closing was photographed. The cell migration rate was calculated as described in “Materials and methods” section. *Bars*, SD. **P < 0.01, BDNF-treated vs. control. ##*P* < 0.01, LY294002 pre-treated or PD98059 pre-treated vs. BDNF-treated. **b** Migration assay was performed as described in the “Materials and methods” section. Representative fields of migrating cells under microscope were shown upper of the figure. The cells that migrated to the underside of the inserts were counted, and Student’s *t* test was done. *Bars*, SD. **P < 0.01, BDNF-treated vs. control. #*P* < 0.05, LY294002 pre-treated or PD98059 pre-treated vs. BDNF-treated. **c** Invasion assay was performed as described in the “Materials and methods” section. Representative fields of invading cells under microscope were shown upper of the figure. The cells that invaded to the underside of the inserts were counted, and Student’s *t* test was done. *Bars*, SD. **P < 0.01, BDNF-treated vs. control. ##*P* < 0.01, LY294002 pre-treated or PD98059 pre-treated vs. BDNF-treated. **d** TB3 cells were pre-treated with PI3K inhibitor LY294002 or MAPK inhibitor PD98059 for 1 h followed by the BDNF treatment for 1 h, and then harvested. Total protein was extracted for Western blotting

The result from scratch wound healing assay showed that the cell migration rates were 54.9 % in the control cells, 93.0 % in the BDNF-treated cells, 54.9 % in the BDNF + LY294002-treated cells, and 62.2 % in the BDNF + PD98059-treated cells (Fig. 3a). BDNF-induced increase of gap closing was blocked by either LY294002 (*P* < 0.01) or PD98059 (*P* < 0.01) in TB3 cells (Fig. 3a). We also used the Boyden chamber migration assay to study the pathway(s) that mediated the BDNF/TrkB-increased TB3 cell migration. The Boyden chamber migration assay was done as described in “Materials and methods” section. The cells that migrated to the underside of the inserts were counted and photographed (Fig. 3b). BDNF-induced increase of migrating cells was reduced by pre-treatment with either LY294002 (*P* < 0.05) or PD98059 (*P* < 0.05) (Fig. 3b).

The pathway(s) that mediated the BDNF/TrkB-increased TB3 cell invasion was tested using a Boyden chamber invasion assay. The Boyden chamber invasion assay was done as described in “Materials and methods” section. The cells that invaded to the underside of the inserts were counted and photographed (Fig. 3c). BDNF-induced increase of invading cells was reduced by pre-treatment with either LY294002 (*P* < 0.01) or PD98059 (*P* < 0.01) (Fig. 3c).

We observed the effects of LY294002 and PD98059 on P-Akt(Ser473) and P-Erk(Thr202/Tyr204) expressions by Western blotting. The results showed that the BDNF/TrkB-induced increase of P-Akt(Ser473) was blocked by LY294002, while PD98059 did not inhibit the expression of P-Akt (Fig. 3d). And the BDNF/TrkB-induced increase of P-Erk(Thr202/Tyr204) was blocked by PD98059, while LY294002 did not inhibit the expression of P-Erk(Thr202/Tyr204) (Fig. 3d).

These data indicated that both PI3K pathway and MAPK pathway mediated BDNF/TrkB effect on TB3 cell migration and invasion.

### Akt and mTOR mediated the BDNF/TrkB-induced increases of cell migration and invasion

**Fig. 4:**
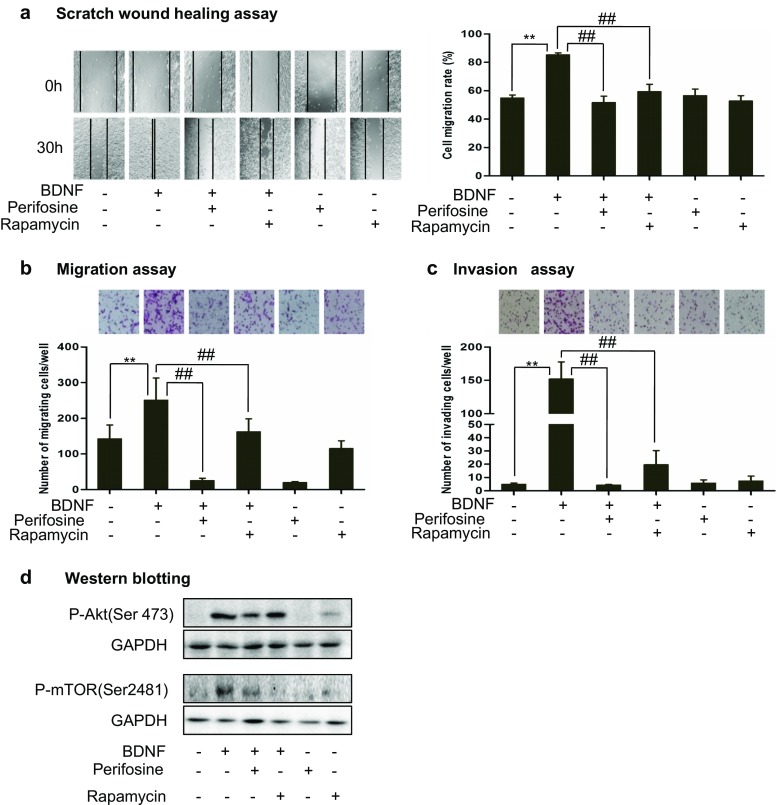
The molecular targets that mediate BDNF/TrkB effects on TB3 cell migration and invasion in vitro. **a** TB3 cells cultured in the absence of TET were scratched with a 200-μl pipette tip across the center of the well; then cells were pre-treated with Akt inhibitor perifosine, or mTOR inhibitor rapamycin for 1 h followed by the BDNF treatment for 30 h. Gap closing was photographed. The cell migration rate was calculated as described in “Materials and methods” section. *Bars*, SD. **P < 0.01, BDNF-treated vs. control. ##*P* < 0.01, Akt or mTOR pre-treated vs. BDNF-treated. **b** Migration assay was performed as described in the “Materials and methods” section. Representative fields of migrating cells under microscope were shown upper of the figure. The cells that migrated to the underside of the inserts were counted, and Student’s *t* test was done. *Bars*, SD. **P < 0.01, BDNF-treated vs. control. ##*P* < 0.01, Akt or mTOR pre-treated vs. BDNF-treated. **c** Invasion assay was performed as described in the “Materials and methods” section. Representative fields of invading cells under microscope were shown upper of the figure. The cells that invaded to the underside of the inserts were counted, and Student’s *t* test was done. *Bars*, SD. **P < 0.01, BDNF-treated vs. control. ##*P* < 0.01, Akt or mTOR pre-treated vs. BDNF-treated. **d** TB3 cells were pre-treated with Akt inhibitor perifosine, or mTOR inhibitor rapamycin for 1 h followed by the BDNF treatment for 1 h, and then harvested. Total protein was extracted for Western blotting

As we have reported that the PI3K/Akt signal transduction pathway mediated the BDNF/TrkB-induced chemo-resistance in NB [9–11], and this study showed that the PI3K pathway also mediated the BDNF/TrkB-increased migration and invasion, we further studied whether or not Akt and its downstream targets mTOR were involved in BDNF/TrkB-induced increases of cell migration and invasion. We pre-treated TB3 cells with Akt inhibitor perifosine or mTOR inhibitor rapamycin for 1 h, and then treated with BDNF for 30 h in scratch wound healing assay, 6 h in Boyden chamber migration assay, or 24 h in Boyden chamber invasion assay. The result from the scratch wound healing assay showed that the cell migration rates were 54.9 % in the control cells, 85.3 % in the BDNF-treated cells, 51.6 % in the BDNF + perifosine-treated cells, and 59.3 % in the BDNF + rapamycin-treated cells (Fig. 4a). BDNF-induced increase of gap closing was blocked by either perifosine (*P* < 0.01) or rapamycin (*P* < 0.01) in TB3 cells (Fig. 4a). For the cell migration evaluated by the Boyden chamber migration assay, we found that BDNF-induced increase of migrating cells was reduced by pre-treatment with either perifosine (*P* < 0.01) or rapamycin (*P* < 0.01) (Fig. 4b). For the cell invasion evaluated by the Boyden chamber invasion assay, we found that BDNF-induced increase of invading cells was also reduced by pre-treatment with either perifosine (*P* < 0.01) or rapamycin (*P* < 0.01) (Fig. 4c).

We also observed the effects of perifosine and rapamycin on P-Akt(Ser473) and P-mTOR(Ser2481) expressions by Western blotting. The results showed that the BDNF/TrkB-induced increase of P-Akt(Ser473) was blocked by perifosine, while rapamycin did not inhibit the expression of P-Akt (Fig. 4d). And the BDNF/TrkB-induced increase of P-mTOR(Ser2481) was blocked by both perifosine and rapamycin (Fig. 4d).

These data indicated that both Akt and mTOR mediated BDNF/TrkB effect on TB3 cell migration and invasion.

## Discussion

In the present study, we indentified that BDNF/TrkB increased TB3 cell metastasis in vitro and in vivo. Inhibitors of PI3K pathway, MAPK pathway, Akt, and mTOR blocked the BDNF/TrkB-increased cell migration and invasion in vitro.

BDNF is needed for the development and maintenance of peripheral sympathetic and neural crest-derived sensory neurons [17–19]. Binding of BDNF to its tyrosine kinase receptor TrkB activates PI3K, MAPK, and PLC-γ pathways [9, 17–18]. BDNF/TrkB has been reported to be highly expressed in many malignancies and associated with poor prognosis [20–24]. In NB, BDNF/TrkB expressions were found in tumors of patients with unfavorable prognosis [6], and BDNF/TrkB promoted NB cell survival and proliferation, and induced resistance to chemo-therapy in vitro and in vivo [25–27]. Our previous studies have indicated that BDNF/TrkB induced chemo-resistance via PI3K/Akt pathway and MAPK pathway [9–11]; we and others have reported that inhibition of TrkB enhanced chemotherapeutic efficacy in NB in vitro and in vivo [13, 28]. Studies also found that BDNF/TrkB increased metastasis in many malignancies, such as pancreatic ductal carcinoma [29], prostate cancer [30], lung cancer [31], hepatocellular carcinoma [21, 32], colorectal cancer [33–34], gastric cancer [35], choriocarcinoma [36], ovarian cancer [37], and multiple myeloma [38]. Some of these reports showed that blocking TrkB or BDNF suppressed the BDNF/TrkB-induced metastasis [21, 34, 36]. Studies have shown that BDNF/TrkB mediated NB cell invasiveness, and Douma S. and Geiger TR. et al. studied the mechanisms of BDNF/TrkB-induced cell invasiveness [15–16, 39–40]. Douma S. et al. reported that TrkB increased rat intestinal epithelial (RIE) cell aggressiveness via suppression of anoikis (apoptosis resulting from loss of cell-matrix interactions), and their results showed that TrkB activated PI3K/PKB signaling, which contributed to anoikis resistance [39]. Geiger TR et al. showed that TrkB increased NB cell aggressiveness via suppression of anoikis; further, they identified that kinase activity, but not adhesion domains, was required for TrkB-induced anoikis suppression in vitro and tumor metastatic capacity in vivo [40]. But further mechanisms have not been shown in their reports. Hecht M. et al. focused their studies on HGF (hepatocyte growth factor) and its receptor c-Met, and their results showed that TrkB up-regulated HGF and c-Met expression to promote NB cell invasive capability [15]. Cimmino F. et al. studied the role of galectin-1 in TrkB-mediated invasiveness in NB cells. They found that activation of TrkB up-regulated galectin-1 expression, and knockdown of galectin-1 mRNA expression or use galectin-1 inhibitor reduced the migratory and invasive capacity of NB cells [16]. In our present study, we explored the role of BDNF/TrkB in NB cell metastasis in vitro and in vivo and further studied the pathways that mediated BDNF/TrkB effect on NB cell migration and invasion.

Our results showed that BDNF activation of TrkB increased the TB3 cell migration and invasion in vitro, and metastasis was significantly increased in TrkB-expressing TB3 tumors compared to that in non-TrkB-expressing TB3 tumors in vivo. These results were similar to Mineyoshi Aoyama and others’ reports in which BDNF/TrkB promoted NB cell invasive activity [6, 15–16, 40]. Although there are studies that focus on the mechanisms of BDNF/TrkB-mediated NB cell metastasis [15–16, 40], the signal transduction pathways that mediated the invasive activity were not fully investigated. Douma S. et al. have reported that TrkB activated PI3K/PKB signaling, which contributed to anoikis resistance in RIE cells [39]. In our present study, we found that suppression of PI3K and MAPK pathways inhibited BDNF/TrkB-increased migration or invasion in TB3 cells, and blocked BDNF/TrkB-induced increase of P-Akt(Ser473) and P-Erk(Thr202/Tyr204) (Fig. 3a–d). Furthermore, inhibitors for Akt and mTOR, two downstream targets in PI3K pathway [26], could also block BDNF/TrkB-increased cell migration and invasion, and blocked BDNF/TrkB-induced increase of P-Akt(Ser473) and P-mTOR(Ser2481) (Fig. 4a–d). Our results showed that inhibitor of mTOR (rapamycin) suppressed the BDNF/TrkB-increased migration and invasion in TB3 cells. This result is differed from Douma S.’s study in which rapamycin did not contribute to TrkB-mediated anoikis resistance in RIE cells [39]. These may suggest that although mTOR is not involved in TrkB-mediated anoikis resistance in RIE cells, it may involve in other key processes during NB cell migration and invasion. Further studies are needed to explain this.

Epithelial mesenchymal transition (EMT) is the conversion of epithelial cells to mesenchymal cells, which is considered to be associated with an increase of cell migration and invasion capacity, as well as resistance to anoikis/apoptosis. EMT was reported to be accompanied by an increase of N-cadherin and Slug, or by a decrease of E-cadherin [41]. In our present study, we investigated the expressions of N-cadherin, E-cadherin, and Slug under the BDNF treatment. We found that the BDNF treatment of TrkB-expressing TB3 cells increased E-cadherin expression, decreased Slug expression, but had no effect on N-cadherin expression within the time points we tested (Suppl. Fig. 1). Inhibitors for PI3K, MAPK, Akt, and mTOR could individually attenuate the BDNF/TrkB-induced increase of E-cadherin or decrease of Slug. These inhibitors had no effect on N-cadherin expression (Suppl. Fig. 2). As our results are different from some reports [41], so further studies are needed to investigate whether or not E-cadherin and Slug are involved in the BDNF/TrkB-induced migration or invasion, and if they are, how do they affect the migration and invasion? Recent reports show that EMT is not required for metastasis in breast cancer and pancreatic cancer [42, 43], so the role of EMT during metastasis in different types of cancers may vary.

Our study provided evidence that BDNF/TrkB increased migration and invasion of TB3 cells through PI3K/Akt and MAPK pathways; together with previous studies that BDNF/TrkB mediated chemo-resistance through PI3K/Akt pathway in the treatment of NB patients [9–14], we believe that BDNF/TrkB and its downstream targets will play very important roles in the treatment of high-risk NB patients, who are suffering from metastasis or/and chemo-resistance. Also, there are some limitations in our present study, such as no validation of the results in clinical tumor samples, only one cell line used, and no more inhibitors targeting BDNF/TrkB signaling pathways. In the future, we will try to finish these studies to fully elucidate the effect of BDNF/TrkB on NB metastasis.

Above all, our study showed that BDNF/TrkB increased TB3 cell migration and invasion through PI3K/Akt/mTOR and MAPK pathways. Based on these findings, we may provide new potential molecular targets for the treatment of NB metastasis.

## Electronic supplementary material

Suppl. Fig. 1 The effect of BDNF/TrkB on the expressions of N-cadherin, E-cadherin and Slug. TrkB-expressing TB3 cells were treated with BDNF (100 ng/ml) for 15 min, 1 h, 2 h, then harvested. Western blotting was performed to detect the expressions of N-cadherin, E-cadherin, and Slug (1:1000 dilution, Cell Signaling Tech.). GAPDH (1:10,000 dilution, Kangchen bio-tech) was used as the loading control. (PDF 80.7 kb)

Suppl. Fig. 2 The effect of PI3K, MAPK, Akt, and mTOR inhibitors on the BDNF/TrkB-induced changes of N-cadherin, E-cadherin, and Slug. TrkB-expressing TB3 cells were pre-treated with each of the inhibitors for 1 h (LY294002, 10 μM; PD98059, 10 μM; perifosine, 5 μM; rapamycin, 100 nM) followed by BDNF(100 ng/ml, 1 h) treatment. Cells were harvested and Western blotting was performed to detect the expressions of N-cadherin, E-cadherin, and Slug (1:1000 dilution, Cell Signaling Tech.), GAPDH (1:10,000 dilution, Kangchen bio-tech) was used as the loading control. (PDF 111 kb)

## References

[1] Brodeur GM, Pritchard J, Berthold F, Carlsen NL, Castel V, Castelberry RP (1993). Revisions of the international criteria for neuroblastoma diagnosis, staging, and response to treatment. J Clin Oncol.

[2] Maris JM, Matthay KK (1999). Molecular biology of neuroblastoma. J Clin Oncol.

[3] Castel V, Grau E, Noguera R, Martinez F (2007). Molecular biology of neuroblastoma. Clin Transl Oncol.

[4] Brodeur GM (2003). Neuroblastoma: biological insights into a clinical enigma. Nat Rev Cancer.

[5] Maris JM, Hogarty MD, Bagatell R, Cohn SL (2007). Neuroblastoma. Lancet.

[6] Aoyama M, Asai K, Shishikura T, Kawamoto T, Miyachi T, Yokoi T (2001). Human neuroblastomas with unfavorable biologies express high levels of brain-derived neurotrophic factor mRNA and a variety of its variants. Cancer Lett.

[7] Nakagawara A, Arima-Nakagawara M, Scavarda NJ, Azar CG, Cantor AB, Brodeur GM (1993). Association between high levels of expression of the TRK gene and favorable outcome in human neuroblastoma. N Engl J Med.

[8] Eggert A, Grotzer MA, Zhao H, Brodeur GM, Evans AE (2001). Expression of the neurotrophin-receptor TrkB predicts outcome in nephroblastomas: results of a pilot-study. Klin Padiatr.

[9] Jaboin J, Kim CJ, Kaplan DR, Thiele CJ (2002). Brain-derived neurotrophic factor activation of TrkB protects neuroblastoma cells from chemotherapy-induced apoptosis via phosphatidylinositol 3'-kinase pathway. Cancer Res.

[10] Li Z, Jaboin J, Dennis PA, Thiele CJ (2005). Genetic and pharmacologic identification of Akt as a mediator of brain-derived neurotrophic factor/TrkB rescue of neuroblastoma cells from chemotherapy-induced cell death. Cancer Res.

[11] Li Z, Oh DY, Nakamura K, Thiele CJ (2011). Perifosine-induced inhibition of Akt attenuates brain-derived neurotrophic factor/TrkB-induced chemoresistance in neuroblastoma in vivo. Cancer.

[12] Ho R, Eggert A, Hishiki T, Minturn JE, Ikegaki N, Foster P (2002). Resistance to chemotherapy mediated by TrkB in neuroblastomas. Cancer Res.

[13] Croucher JL, Iyer R, Li N, Molteni V, Loren J, Gordon WP (2015). TrkB inhibition by GNF-4256 slows growth and enhances chemotherapeutic efficacy in neuroblastoma xenografts. Cancer Chemother Pharmacol.

[14] Iyer R, Varela CR, Minturn JE, Ho R, Simpson AM, Light JE (2012). AZ64 inhibits TrkB and enhances the efficacy of chemotherapy and local radiation in neuroblastoma xenografts. Cancer Chemother Pharmacol.

[15] Hecht M, Schulte JH, Eggert A, Wilting J, Schweigerer L (2005). The neurotrophin receptor TrkB cooperates with c-Met in enhancing neuroblastoma invasiveness. Carcinogenesis.

[16] Cimmino F, Schulte JH, Zollo M, Koster J, Versteeg R, Iolascon A (2009). Galectin-1 is a major effector of TrkB-mediated neuroblastoma aggressiveness. Oncogene.

[17] Ninan I (2014). Synaptic regulation of affective behaviors; role of BDNF. Neuropharmacology.

[18] Thiele CJ, Li Z, McKee AE (2009). On Trk--the TrkB signal transduction pathway is an increasingly important target in cancer biology. Clin Cancer Res.

[19] Yoshii A, Constantine-Paton M (2010). Postsynaptic BDNF-TrkB signaling in synapse maturation, plasticity, and disease. Dev Neurobiol.

[20] Vanhecke E, Adriaenssens E, Verbeke S, Meignan S, Germain E, Berteaux N (2011). Brain-derived neurotrophic factor and neurotrophin-4/5 are expressed in breast cancer and can be targeted to inhibit tumor cell survival. Clin Cancer Res.

[21] Guo D, Hou X, Zhang H, Sun W, Zhu L, Liang J (2011). More expressions of BDNF and TrkB in multiple hepatocellular carcinoma and anti-BDNF or K252a induced apoptosis, supressed invasion of HepG2 and HCCLM3 cells. J Exp Clin Cancer Res.

[22] Brunetto de Farias C, Rosemberg DB, Heinen TE, Koehler-Santos P, Abujamra AL, Kapczinski F (2010). BDNF/TrkB content and interaction with gastrin-releasing peptide receptor blockade in colorectal cancer. Oncology.

[23] Jia S, Wang W, Hu Z, Shan C, Wang L, Wu B (2015). BDNF mediated TrkB activation contributes to the EMT progression and the poor prognosis in human salivary adenoid cystic carcinoma. Oral Oncol.

[24] Okamura K, Harada T, Wang S, Ijichi K, Furuyama K, Koga T (2012). Expression of TrkB and BDNF is associated with poor prognosis in non-small cell lung cancer. Lung Cancer.

[25] Czarnecka M, Trinh E, Lu C, Kuan-Celarier A, Galli S, Hong SH (2015). Neuropeptide Y receptor Y5 as an inducible pro-survival factor in neuroblastoma: implications for tumor chemoresistance. Oncogene.

[26] Liu P, Cheng H, Roberts TM, Zhao JJ (2009). Targeting the phosphoinositide 3-kinase pathway in cancer. Nat Rev Drug Discov.

[27] Matsumoto K, Wada RK, Yamashiro JM, Kaplan DR, Thiele CJ (1995). Expression of brain-derived neurotrophic factor and p145TrkB affects survival, differentiation, and invasiveness of human neuroblastoma cells. Cancer Res.

[28] Li Z, Zhang Y, Tong Y, Tong J, Thiele CJ (2015). Trk inhibitor attenuates the BDNF/TrkB-induced protection of neuroblastoma cells from etoposide in vitro and in vivo. Cancer Biol Ther.

[29] Miknyoczki SJ, Lang D, Huang L, Klein-Szanto AJ, Dionne CA, Ruggeri BA (1999). Neurotrophins and Trk receptors in human pancreatic ductal adenocarcinoma: expression patterns and effects on in vitro invasive behavior. Int J Cancer.

[30] Montano X, Djamgoz MB (2004). Epidermal growth factor, neurotrophins and the metastatic cascade in prostate cancer. FEBS Lett.

[31] Ricci A, Greco S, Mariotta S, Felici L, Bronzetti E, Cavazzana A (2001). Neurotrophins and neurotrophin receptors in human lung cancer. Am J Respir Cell Mol Biol.

[32] Yang ZF, Ho DW, Lam CT, Luk JM, Lum CT, Yu WC (2005). Identification of brain-derived neurotrophic factor as a novel functional protein in hepatocellular carcinoma. Cancer Res.

[33] Fujikawa H, Tanaka K, Toiyama Y, Saigusa S, Inoue Y, Uchida K (2012). High TrkB expression levels are associated with poor prognosis and EMT induction in colorectal cancer cells. J Gastroenterol.

[34] Tanaka K, Okugawa Y, Toiyama Y, Inoue Y, Saigusa S, Kawamura M (2014). Brain-derived neurotrophic factor (BDNF)-induced tropomyosin-related kinase B (Trk B) signaling is a potential therapeutic target for peritoneal carcinomatosis arising from colorectal cancer. PLoS One.

[35] Okugawa Y, Tanaka K, Inoue Y, Kawamura M, Kawamoto A, Hiro J (2013). Brain-derived neurotrophic factor/tropomyosin-related kinase B pathway in gastric cancer. Br J Cancer.

[36] Kawamura K, Kawamura N, Okamoto N, Manabe M (2013). Suppression of choriocarcinoma invasion and metastasis following blockade of BDNF/TrkB signaling. Cancer Med.

[37] Au CW, Siu MK, Liao X, Wong ES, Ngan HY, Tam KF (2009). Tyrosine kinase B receptor and BDNF expression in ovarian cancers—effect on cell migration, angiogenesis and clinical outcome. Cancer Lett.

[38] Hu Y, Sun CY, Wang HF, Guo T, Wei WN, Wang YD (2006). Brain-derived neurotrophic factor promotes growth and migration of multiple myeloma cells. Cancer Genet Cytogenet.

[39] Douma S, Van Laar T, Zevenhoven J, Meuwissen R, Van Garderen E, Peeper DS (2004). Suppression of anoikis and induction of metastasis by the neurotrophic receptor TrkB. Nature.

[40] Geiger TR, Peeper DS (2007). Critical role for TrkB kinase function in anoikis suppression, tumorigenesis, and metastasis. Cancer Res.

[41] Lee JM, Dedhar S, Kalluri R, Thompson EW (2006). The epithelial-mesenchymal transition: new insights in signaling, development, and disease. J Cell Biol.

[42] Fischer KR, Durrans A, Lee S, Sheng J, Li F, Wong ST (2015). Epithelial-to-mesenchymal transition is not required for lung metastasis but contributes to chemoresistance. Nature.

[43] Zheng X, Carstens JL, Kim J, Scheible M, Kaye J, Sugimoto H (2015). Epithelial-to-mesenchymal transition is dispensable for metastasis but induces chemoresistance in pancreatic cancer. Nature.

