# Supplementary figures and images for: PI3K and MAPK pathways mediate the BDNF/TrkB-increased metastasis in neuroblastoma
Source: Tumour Biol. 2016 Oct 17;37(12):16227–36. doi: 10.1007/s13277-016-5433-z (PMC5250655; doi:10.1007/s13277-016-5433-z)

Suppl. Fig. 1

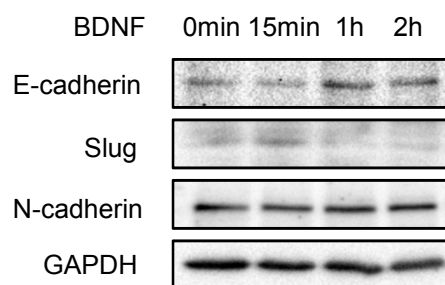

Supplement: Supplementary file 1 — The effect of BDNF/TrkB on the expressions of N-cadherin, E-cadherin and Slug. TrkB-expressing TB3 cells were treated with BDNF (100 ng/ml) for 15 min, 1 h, 2 h, then harvested. Western blotting was performed to detect the expressions of N-cadherin, E-cadherin, and Slug (1:1000 dilution, Cell Signaling Tech.). GAPDH (1:10,000 dilution, Kangchen bio-tech) was used as the loading control. (PDF 80.7 kb) [file 13277_2016_5433_MOESM1_ESM.pdf]

Suppl. Fig. 2

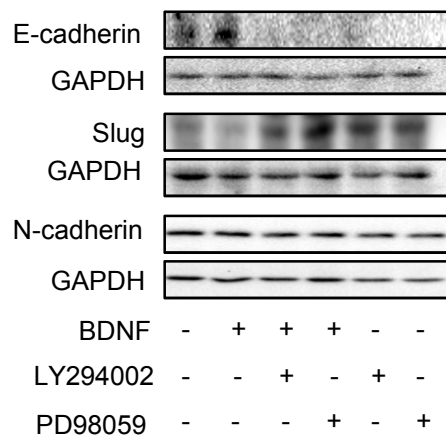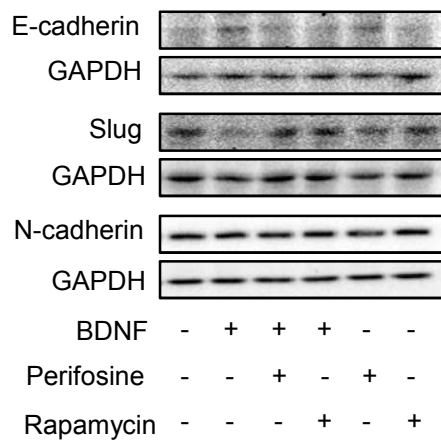

Supplement: Supplementary file 2 — The effect of PI3K, MAPK, Akt, and mTOR inhibitors on the BDNF/TrkB-induced changes of N-cadherin, E-cadherin, and Slug. TrkB-expressing TB3 cells were pre-treated with each of the inhibitors for 1 h (LY294002, 10 μM; PD98059, 10 μM; perifosine, 5 μM; rapamycin, 100 nM) followed by BDNF(100 ng/ml, 1 h) treatment. Cells were harvested and Western blotting was performed to detect the expressions of N-cadherin, E-cadherin, and Slug (1:1000 dilution, Cell Signaling Tech.), GAPDH (1:10,000 dilution, Kangchen bio-tech) was used as the loading control. (PDF 111 kb) [file 13277_2016_5433_MOESM2_ESM.pdf]
